# Vaccination Intention against COVID-19 among the Unvaccinated in Jordan during the Early Phase of the Vaccination Drive: A Cross-Sectional Survey

**DOI:** 10.3390/vaccines10071159

**Published:** 2022-07-21

**Authors:** Ilias Mahmud, Mahmudul Hassan Al Imam, Divya Vinnakota, Khalid A. Kheirallah, Mahmoud F. Jaber, Adil Abalkhail, Ibrahim Alasqah, Thamer Alslamah, Russell Kabir

**Affiliations:** 1 Department of Public Health, College of Public Health and Health Informatics, Qassim University, Al Bukairiyah 52741, Saudi Arabia; i.emdadulhaque@qu.edu.sa (I.M.); mf.jaber@qu.edu.sa (M.F.J.); abalkhail@qu.edu.sa (A.A.); i.alasqah@qu.edu.sa (I.A.); 4037@qu.edu.sa (T.A.); 2 School of Health, Medical and Applied Sciences, Central Queensland University, Rockhampton, QLD 4701, Australia; m.alimam@cqu.edu.au; 3 Central Queensland Public Health Unit, Central Queensland Hospital and Health Service, Rockhampton, QLD 4700, Australia; 4 Department of Public Health and Nursing, University of Sunderland, London E14 9SG, UK; divya.vinnakota@sunderland.ac.uk; 5 Department of Public Health, Medical School of Jordan University of Science and Technology, P.O. Box 3030, Irbid 22110, Jordan; kkheiral@gmail.com; 6 School of Allied Health, Faculty of Health, Education, Medicine and Social Care, Anglia Ruskin University, Chelmsford CM1 1SQ, UK

**Keywords:** COVID-19, SARS-CoV-2, vaccine hesitancy, health belief model, Jordan

## Abstract

Objective: This study assessed the intention and predictors of accepting the corona virus disease 2019 (COVID-19) vaccine in Jordan. Method: A national-level online survey was conducted among adults (≥18 years) in Jordan between June and September 2021. Descriptive analyses were performed to report vaccination intent. In addition, bivariate and multivariate logistic regression analyses were done to evaluate the association between vaccination intent and its predictors. Results: A total of 2307 adults participated. Most of them (83.7%) expressed an intention to receive a COVID-19 vaccine. Their vaccination intention was significantly (*p* < 0.001) associated with male gender (aOR: 2.6), residence in the Amman region (aOR: 51.8), and no history of COVID-19 infection (aOR: 6.0). In contrast, individuals aged 50-64 years (aOR: 0.2, *p* < 0.001), Jordanians (aOR: 0.7, *p* = 0.038), and those with an occupation designated as “other” (unemployed, general workers, housewives) (aOR: 0.2, *p* < 0.001) were less likely to have a positive vaccination intent. Among the health belief model constructs, perceived future (aOR: 2.8) and present (aOR: 5.0) susceptibility to COVID-19 infection; severity of complications (aOR: 9.9); and benefits (aOR: 100.8) were significantly (*p* < 0.001) associated with a higher likelihood of having a vaccination intent. On the other hand, individuals who are concerned about the efficacy (aOR: 0.2) and side effects (aOR: 0.2) of the vaccine were less likely to have a positive vaccination intent (*p* < 0.001). Conclusion: Despite having high rates of intention to receive a COVID-19 vaccine, Jordanians, older adults and housewives, general workers and unemployed individuals were less likely to be vaccinated. These findings highlight that need-based public health campaigns are necessary to ensure maximum COVID-19 vaccination uptake in Jordan.

## 1. Introduction

Coronavirus disease-2019 (COVID-19) is caused by the severe acute respiratory syndrome coronavirus 2 (SARS-CoV-2) [1]. The disease was first reported in December 2019 in Wuhan, China [2]. The disease then spread widely across the world, posing a serious humanitarian and economic burden, as well as having a detrimental effect on healthcare systems [3]. According to the World Health Organization (WHO), as of 11 February 2022, more than 404.9 million COVID-19 cases and 5.7 million COVID-19-related deaths were reported globally [4]. In Jordan, 1,417,890 COVID-19 cases and 13,431 COVID-19-related deaths had been reported by 11 February 2022 [4]. 

Effective vaccines, which can help in reducing transmission, hospital admissions, and the demand for intensive care, are a critical tool for controlling the continuing COVID-19 epidemic [5]. As of 27 June 2022, 44.5% of the population has received at least two doses of a COVID-19 vaccine in Jordan [4].

Vaccine hesitancy is people’s unwillingness or complete refusal to vaccinate, even when vaccines are available [6]. The WHO identified vaccine hesitancy as being among the top 10 threats to global health [6]. Various nations have different acceptance rates for COVID-19 vaccination, ranging from less than 55% in Russia to 90% in China [7]. Another study conducted in Saudi Arabia stated that more than half (58%) of the participants intended to receive a COVID-19 vaccine [8]. A similar study conducted in Bangladesh reported that 25.5% said they would definitely receive the vaccine and 43% said they would probably receive the vaccine [9]. 

Vaccine hesitancy is often the result of a poor understanding of the actual dangers from a disease, a lack of confidence in the available vaccines or the authority, or inconvenience in accessing the vaccines [6]. Furthermore, other unmeasurable influences that vary by context, time, place, and vaccination type may complicate the situation [10]. A multi-country study on potential acceptance of a COVID-19 vaccine [7] reported that older age groups, female gender, higher level of education, higher level of income, higher level of trust in the government, and a medium to a high number of cases and fatality rates in the country were positively associated with vaccine acceptance. Meanwhile, in Saudi Arabia, age, occupation, and previous vaccination status were significantly related to vaccine uptake [8]. In Jordan, in December 2020, it was estimated that 29.4% of eligible individuals were willing to receive a COVID-19 vaccine when available [11]. 

Few studies had been conducted in Jordan with a focus on investigating public perception and hesitancy toward any possible new COVID-19 vaccine before the availability of such a vaccine in the country [11,12,13]. However, vaccination intent may change over time [7]. Therefore, it is important to see whether people’s attitudes toward the vaccine changed in Jordan while the vaccination program was running. Furthermore, none of the previous studies used the Health Belief Model (HBM) to investigate vaccine hesitancy. The HBM is a conceptual framework for explaining, predicting, and influencing individual or group behavior related to health issues. This model explains that actions involving health issues require sufficient motivation (e.g., illness or health concern), a perceived threat, a perceived serious health problem/complication caused by an illness, perceived benefits, belief that following health recommendations will help reduce the perceived threats, and the conviction that the benefits outweigh the costs [14,15]. Recently, the HBM model was used to predict COVID-19 vaccination intent in different countries [8,9,16,17,18]. In this context, the aim of this study was to investigate COVID-19 vaccination intent among the general population in Jordan and to explore the factors, including the HBM constructs, which are associated with positive vaccination intent. 

## 2. Methods

### 2.1. Study Design and Participant

We conducted a nationwide, online, cross-sectional survey in Jordan between June and September 2021. Adult (≥18 years) citizens or residents of Jordan who were not yet fully vaccinated were considered eligible for this study. To disseminate our online questionnaire, we used all commonly used social media platforms in Jordan such as Facebook, Instagram, WhatsApp, and Twitter. We also distributed a short message containing the survey link and participation invitation through a telecommunication service provider in Jordan. Every individual who accessed our online survey link was requested to forward it to their network. A total of 2307 adults aged 18 years or more and residing in Jordan during the study period participated in this online survey. 

### 2.2. Assessment and Outcomes

We developed our structured questionnaire by reviewing previously published studies that used the HBM to investigate vaccination hesitancy, including hesitancy against a COVID-19 vaccine [8,9,16,17,18,19,20,21,22,23]. 

Through the structured questionnaire, we collected information on socio-demographic variables (age, gender, ethnicity, religion, marital status, education, and occupation), known diagnosis of any chronic diseases, an COVID-19 infection status among participants and their family members, relatives, friends, neighbors, or colleagues. By family members, we meant first-degree relatives, including an individual’s parents, siblings, spouse, and children. 

We assessed our study participants’ COVID-19 vaccination intent using a question, i.e., if a vaccine against COVID-19 infection were available to you, would you take it? Participants were given four response options: definitely not, probably not, probably yes, and definitely yes.

In addition, we asked questions to assess the following HBM constructs: perceived susceptibility to COVID-19 infection (three questions), perceived severity of COVID-19 infection (three questions), perceived benefits of a COVID-19 vaccine (two questions), perceived barriers to getting a vaccination against COVID-19 (five questions), and cues to action (two questions). We used simplified response options for these questions, i.e., agree/disagree. 

### 2.3. Statistical Analysis

Data were analyzed using the R Studio 4.1.2 (R Foundation for Statistical Computing, Vienna, Austria). The primary study variable of interest was the intention to receive a COVID-19 vaccine, which had four categories, i.e., ‘definitely not’, ‘probably not’, ‘probably yes’, and ‘definitely yes’. For our analyses, we re-categorized these into dichotomous responses, i.e., ‘yes’ and ‘no’. Additionally, age groups, education, regions, and nationality were recoded via dichotomous responses. Descriptive analyses were performed to visualize the proportions of the study variables. Chi-square tests were used to determine the factors associated with intention to receive COVID-19 vaccines. Bivariate and multivariate logistic regression analyses were computed to evaluate the strength of association. *P* values less than 0.05 were considered statistically significant. Variables found to be significant in the bivariate analyses were used in the multivariate regression models. Odds Ratio (OR), adjusted Odds Ratio (aOR), and 95% Confidence Intervals (CIs) were calculated. Similarly, we assessed the association between perceived COVID-19-related health beliefs and intention to receive a COVID-19 vaccine.

Ethical approval for this study was obtained from the Institutional Review Board of the Jordan University of Science and Technology (number: 6/146/2021). Participants were informed about the objectives of the study. They were also informed that participating in this study was completely voluntary and participation/non-participation were not associated with any personal benefit or harm. The first page of the online survey form included the informed consent form. Participants who provided informed consent were allowed to complete the survey. 

## 3. Results

A total of 2307 individuals residing in Jordan participated in the online survey; most belonged to the 18–39 age group (66.5%) and were female (54.5%). The majority of the respondents were Jordanian nationals (84.1%), from Amman (the capital city) (42.4%) and had completed tertiary education (60.9%). Forty-seven per cent of the study participants had been diagnosed with COVID-19 at some point preceding this study, and only 10.4% reported receiving flu vaccine each year. Most of the respondents (83.7%) expressed their intentions to receive the COVID-19 vaccine (Table 1).

Unadjusted analyses found that male gender, 30-39 years age group, higher education (tertiary level), living in Amman, being a non-health professional, Jordanian nationality, no history of COVID-19 infection, and no family history of COVID-19 infection were associated with a positive intention to receive a COVID-19 vaccine. On the other hand, older individuals (40-49 years and 50-64 years age groups) were less likely to have positive intention to receive a vaccine. However, following the adjustment of potential confounders, we found that male (aOR: 2.6; CI: 1.8–3.7), residents of the Amman region (aOR: 51.8; CI: 27.7–104.1), non-health professionals (aOR: 1.6; CI: 0.9–2.8), and individuals without a history COVID-19 infection (aOR: 6.0; CI: 3.3–11.5) had a higher likelihood of having a positive vaccination intent against the COVID-19 when compared to female, other city residents, health professionals and individuals with a history of COVID-19 infection, respectively. In contrast, older individuals (50–64 years; aOR: 0.2; CI: 0.1–0.4), Jordanian nationals (aOR: 0.7; CI: 0.4–1.0), and those with an occupation designated as “other” (unemployed, general workers, housewives) (aOR: 0.2; 0.1–0.3) were less likely to have a positive COVID-19 vaccination intent when compared against individuals aged 18–29 years, non-Jordanian nationals, and health professionals, respectively (Table 1).

Our analyses revealed that more than half of the study participants agreed with the stated susceptibility of contracting COVID-19 (present: 64.5% and future: 50.8%). We found that the majority of the participants believed that COVID-19 complications were serious (76.9%), and they would be very sick if they got infected with the virus (69.2%). Seventy-nine per cent of study participants expressed their confidence in the COVID-19 vaccine in terms of decreasing their risk of getting the disease, while 47.9% were concerned about the efficacy and 53.0% about the safety/side effects of the vaccine. The Halal nature of the vaccine was not of concern for most participants (77.2%). We found that the majority of the study participants intended to receive the vaccine after a large number of people had received it (56.6%), after receiving in-depth information (81.1%), or if the vaccine did not cause any harm to vaccinated people (51.2%) (Table 2). 

Our multivariable logistic regression analyses revealed that respondents who perceived themselves as being susceptible to the virus, perceived COVID-19 as a severe disease, and believed that the COVID-19 vaccine would reduce their risk of getting the disease were more likely to have a positive intent to receive a COVID-19 vaccine. On the other hand, participants who had expressed their concerns about the efficacy of the available vaccine, the safety of the vaccine, and the halal nature of the vaccine were less likely to have a positive intent to receive it. In addition, participants who intended to wait until many other people had received the vaccine to determine if it was safe were less likely to have a positive vaccination intent (Table 2). 

## 4. Discussion

The rates of intent to receive a vaccine against COVID-19 in Jordan are reported in this article, as are the ways in which health belief model (HBM) constructs can help predict this intent. This study revealed that most adults in Jordan have the intention to receive a COVID-19 vaccine. The positive predictors of intention to receive a vaccine were male gender, residence in the Amman region, and absence of COVID-19 infection. In contrast, Jordanian nationals, older adults, and those with an occupation designated as “other” (unemployed, general workers, housewives) were less likely to have a positive intent to get a COVID-19 vaccine. Among the health belief model constructs, perceived susceptibility, perceived severity of COVID-19, and perceived benefits were significantly associated with a higher likelihood of having a positive COVID-19 vaccination intent.

Our study results suggest that individuals aged 50–64 years of age are less likely to have positive vaccination intent compared to those aged 18–29 years. Perhaps older people in Jordan are less outgoing than younger people, and hence, feel less susceptible to contracting the virus. There is contradicting evidence regarding the association between age and vaccine hesitancy. In Singapore, middle-aged and older adults were more hesitant to receive a COVID-19 vaccine [24], whereas a study conducted in Ireland and the United Kingdom [25] reported that younger (18–24 years) people are more hesitant. 

Our study findings suggest that males are more likely to have a positive intent to receive a COVID-19 vaccine than females. This finding is inconsistent with a global survey which reported that men are more likely to be vaccine hesitant compared to women [7]. However, a recent systematic review reported that the majority of studies have found that men have a higher intention to receive a COVID-19 vaccine than women [26], in line with our results.

Our study suggests that Jordanians and residents of cities other than Amman are less likely to have a positive intent to receive a COVID-19 vaccine. Similarly, another study conducted in Bangladesh stated that residents of other cities are significantly less likely than residents of the capital city, Dhaka, to have a willingness to get vaccinated [9]. Perhaps in big cities, people feel more susceptible to the virus than in smaller cities, or perhaps vaccination programs are operating with different intensities in different cities with greater focus in the bigger, more densely populated cities. 

Our study suggests that people who did not have a history of COVID-19 infection are more likely to accept the vaccine. In contrast, Kabir et al. [9] reported that in Bangladesh, people who had previously been infected with COVID-19 were nearly three times more likely than the general population to accept a COVID-19 vaccine. However, the Bangladesh study was conducted at the beginning of the COVID-19 pandemic [9]. At that time, perhaps people had heightened fear of the virus, and hence, people infected with the disease might have experienced higher levels of stigma. People infected with COIVD-19 had negative experiences with their families, their community and even in hospitals from the healthcare workers. These negative experiences might have resulted in greater willingness to get the vaccine among the people who had had the disease in the past in Bangladesh. 

Our analysis of the HBM constructs revealed that those who perceive themselves to be susceptible to SARS-CoV-2 infection, perceive COVID-19 complications as severe, and perceive the benefit of the vaccine against SARS-CoV-2 infection are more likely to have a positive intent to receive a COVID-19 vaccine. Similar findings were reported by studies using the HBM to investigate COVID-19 vaccine hesitancy in Malaysia [16], Hong Kong [27], Bangladesh [9], and Saudi Arabia [8].

In contrast, our results suggest that individuals who are concerned with the efficacy and safety of the vaccine are less likely to have a positive intent. Furthermore, we found that individuals who would get the vaccine only after many in the public had received it, and only after observing that the vaccine did not induce any harm in the vaccinated population, are less likely to have positive vaccination intent. Our findings in this regard concur with reports published from different international contexts [8,9,16,27]. 

In addition, some people are worried that the vaccine is not halal, and therefore, are less likely to get vaccinated. Halal is a term that refers to something that is permissible under Islamic law. Halal typically refers to the ability to eat, drink, or do something in accordance with Islamic law and principles. A recent study on vaccine development in Malaysia revealed a willingness to trust ‘halal’ vaccines. Parents of Malay Muslim children have expressed concerns about the halal status of vaccine ingredients, believing that imported vaccines may contain porcine-derived agents, such as deoxyribonucleic acid (DNA), which is not halal, as Muslims are generally prohibited from using such products, including in medicines [28]. Similar concerns about vaccination against COVID-19 have arisen in Indonesia [29]. However, the WHO clearly stated that COVID-19 vaccines are free from porcine-derived agents, and therefore, are halal [30]. In general, people are receiving conflicting information about COVID-19 vaccines. Social media has played a significant role in the spread of anti-vaccination misinformation and rumors, for example, that vaccines are not halal. Such misinformation has become critical in the ongoing pandemic, causing panic over COVID-19 vaccine safety [31]. Misinformation about the side effects of the available COVID-19 vaccines, rumors, and conspiracy theories regarding vaccines and the pandemic have had a negative impact on the population’s willingness to get vaccinated [32,33,34]. A study conducted in Hong Kong suggests that COVID-19 vaccine intake is significantly associated with trust in the healthcare system or vaccine manufacturers [27]. Therefore, it is critical for the public health bodies to fight vaccine misinformation, such as that spread via social media, using appropriate strategies to reach misinformed people.

Our study had a larger sample size than other studies investigating COVID-19 vaccine hesitancy in Jordan. However, in light of the following limitations, we advise that our study findings be interpreted with caution: Firstly, equal representation from all socio-demographic groups, regarding, e.g., age, gender, nationality, and region could not be ensured in our study. The use of an online survey might have excluded participants without access to the internet or social media platforms, and thus introduced selection bias. Furthermore, we were unable to know the number of people who received our invitation, and hence, we could not report the non-response rate. Also, we could not verify the vaccination status of participants because of the data collection method employed in this study. However, this method was used in earlier studies on vaccine hesitancy [8,35]. This study could not compare the socio-demographic factors of the respondents and non-respondents, which could be a potential confounding factor. Although we piloted the developed questionnaire, we could not validate the methods and findings of the questionnaire development process. Furthermore, the cross-sectional nature of the study and the sampling methods restrict causal inference and generalization of the study findings, respectively.

## 5. Conclusions

This study highlights a high rate (83.7%) of positive intention to receive a COVID-19 vaccine in Jordan. We found associations between COVID-19 vaccination intent and age, gender, occupation, nationality, and area of residence. Males and those residing in Amman city are more likely to have positive vaccination intent than females and those living in other cities, respectively. Similarly, Jordanian nationals, people aged 50–64 years, and those with an occupation designated as “other” (e.g., unemployed, general workers, housewives) are less likely to have a positive intent compared to non-Jordanian nationals, people aged 18–29, and health professionals, respectively. In addition, people who perceive themselves to be susceptible to SARS-CoV-2 infection or perceive the vaccine as beneficial are more likely to have a positive vaccination intent. In contrast, individuals who have concerns of the efficacy and safety of the vaccine are less likely to have a positive intent. Vaccine hesitancy is a major hindrance to controlling COVID-19 outbreaks. Our findings highlight that need-based public health campaigns targeting all population groups and addressing misinformation about the pandemic and COVID-19 vaccines are necessary to ensure maximum COVID-19 vaccination uptake in Jordan.

## Figures and Tables

**Table 1:** Socio-demographic factors associated with the intention to receive a COVID-19 vaccine in Jordan.

Variables	Levels	Intention to Receive a COVID-19 Vaccine				
		No	Yes	Total	Univariable Analysis	Multivariable Analysis ^a^
		N (Row %)		N (Col. %)	OR (95% CI of OR, *p* Value)	
Age group	18–29	61 (11.9)	452 (88.1)	513 (22.2)	-	-
	30–39	70 (6.9)	951 (93.1)	1021 (44.3)	1.83 (1.28–2.63, *p* = 0.001)	0.98 (0.53–1.81, *p* = 0.960)
	40–49	88 (20.0)	351 (80.0)	439 (19.0)	0.54 (0.38–0.77, *p* = 0.001)	0.81 (0.43–1.54, *p* = 0.526)
	50–64	157 (47.0)	177 (53.0)	334 (14.5)	0.15 (0.11–0.21, *p* < 0.001)	0.20 (0.10–0.41, *p* < 0.001)
						
Sex	Female	233 (18.5)	1024 (81.5)	1257 (54.5)	-	-
	Male	143 (13.6)	906 (86.4)	1049 (45.5)	1.44 (1.15–1.81, *p* = 0.002)	2.56 (1.78–3.72, *p* < 0.001)
Education	Secondary or below	228 (25.3)	674 (74.7)	902 (39.1)	-	-
	Tertiary	148 (10.5)	1257 (89.5)	1405 (60.9)	2.87 (2.29–3.61, *p* < 0.001)	1.01 (0.64–1.58, *p* = 0.973)
Nationality	Non-Jordanian	74 (20.2)	293 (79.8)	367 (15.9)	-	-
	Jordanian	302 (15.6)	1638 (84.4)	1940 (84.1)	1.37 (1.03–1.81, *p* = 0.029)	0.65 (0.44–0.97, *p* = 0.038)
Region	Other cities	362 (27.2)	967 (72.8)	1329 (57.6)	-	-
	Amman	14 (1.4)	964 (98.6)	978 (42.4)	25.78 (15.60-46.43, p < 0.001)	51.78 (27.74-104.05, p < 0.001)
Occupation	Health professionals	30 (12.0)	220 (88.0)	250 (12.7)	-	-
	Non-health professionals	38 (5.6)	644 (94.4)	682 (34.5)	2.31 (1.39–3.81, *p* = 0.001)	1.57 (0.87–2.84, *p* = 0.136)
	Other	230 (32.3)	483 (67.7)	713 (36.1)	0.29 (0.19–0.43, *p* < 0.001)	0.16 (0.08–0.30, *p* < 0.001)
	Student	45 (13.6)	285 (86.4)	330 (16.7)	0.86 (0.52–1.41, *p* = 0.561)	0.53 (0.24–1.13, *p* = 0.102)
Receive flu vaccine every year	Yes	44 (18.3)	197 (81.7)	241 (10.4)	-	-
	No	332 (16.1)	1734 (83.9)	2066 (89.6)	1.17 (0.82–1.64, *p* = 0.385)	-
History of COVID-19 infection	Yes	240 (22.2)	841 (77.8)	1081 (46.9)	–	–
	No	136 (11.1)	1090 (88.9)	1226 (53.1)	2.29 (1.82–2.88, *p* < 0.001)	5.97 (3.30–11.48, *p* < 0.001)
History of COVID-19 infection in the family	Yes	255 (18.1)	1153 (81.9)	1408 (61.0)	–	–
	No	121 (13.5)	778 (86.5)	899 (39.0)	1.42 (1.13–1.80, *p* = 0.003)	1.02 (0.52–1.93, *p* = 0.949)

^a^ All variables found significant in the unadjusted analysis were included in the adjusted model to identify potential predictors of intent to receive a COVID-19 vaccine.

**Table 2:** Health belief model predictors of a positive intention to receive a COVID-19 vaccine in Jordan.

Variables	Levels	Intention to Receive COVID-19 Vaccine				
		No	Yes	Total	Univariable Analysis	Multivariable Analysis ^a^
		N (Row %)		N (Col. %)	OR (95% CI of OR, *p* Value)	
Perceived susceptibility						
Chance of getting COVID-19 in the future is very high	Disagree	301 (26.5)	835 (73.5)	1136 (49.2)	-	-
	Agree	75 (6.4)	1096 (93.6)	1171 (50.8)	5.27 (4.05–6.93, *p* < 0.001)	2.81 (1.84–4.34, *p* < 0.001)
Currently, getting COVID-19 is a strong possibility	Disagree	229 (27.9)	591 (72.1)	820 (35.5)	-	-
	Agree	147 (9.9)	1340 (90.1)	1487 (64.5)	3.53 (2.81–4.45, *p* < 0.001)	5.00 (2.82–9.04, *p* < 0.001)
Perceived severity						
Complications of COVID-19 are very serious	Disagree	191 (35.9)	341 (64.1)	532 (23.1)	-	-
	Agree	185 (10.4)	1590 (89.6)	1775 (76.9)	4.81 (3.81–6.08, *p* < 0.001)	9.93 (5.35–18.76, *p* < 0.001)
I will be very sick if I get COVID-19	Disagree	177 (24.9)	534 (75.1)	711 (30.8)	-	-
	Agree	199 (12.5)	1397 (87.5)	1596 (69.2)	2.33 (1.86–2.92, *p* < 0.001)	0.14 (0.06–0.28, *p* < 0.001)
Perceived benefits						
Vaccination will decrease my chances of getting COVID-19	Disagree	307 (63.7)	175 (36.3)	482 (20.9)	-	-
	Agree	69 (3.8)	1756 (96.2)	1825 (79.1)	44.65 (33.14–60.89, *p* < 0.001)	100.77 (57.09–186.95, *p* < 0.001)
Perceived barriers						
Concerned about the efficacy of the vaccine	Disagree	17 (1.4)	1185 (98.6)	1202 (52.1)	-	-
	Agree	359 (32.5)	746 (67.5)	1105 (47.9)	0.03 (0.02–0.05, *p* < 0.001)	0.22 (0.09–0.46, *p* < 0.001)
Concerned about the safety/side effects of the vaccine	Disagree	17 (1.6)	1068 (98.4)	1085 (47.0)	-	-
	Agree	359 (29.4)	863 (70.6)	1222 (53.0)	0.04 (0.02–0.06, *p* < 0.001)	0.19 (0.08–0.43, *p* < 0.001)
Concerned about the halal nature of the vaccine	Disagree	194 (10.9)	1587 (89.1)	1781 (77.2)	-	-
	Agree	182 (34.6)	344 (65.4)	526 (22.8)	0.23 (0.18–0.29, *p* < 0.001)	2.24 (1.31–3.92, *p* = 0.004)
Cues to action						
Will get vaccine after receiving complete information	Disagree	64 (14.7)	371 (85.3)	435 (18.9)	-	-
	Agree	312 (16.7)	1560 (83.3)	1872 (81.1)	0.86 (0.64–1.15, *p* = 0.320)	-
Will get vaccine if it is first accepted by many people	Disagree	80 (8.0)	922 (92.0)	1002 (43.4)	-	-
	Agree	296 (22.7)	1009 (77.3)	1305 (56.6)	0.30 (0.23–0.38, *p* < 0.001)	0.29 (0.16–0.53, *p* < 0.001)
Will get vaccine if it does not cause undue problems to vaccinated people	Disagree	64 (5.7)	1062 (94.3)	1126 (48.8)	-	-
	Agree	312 (26.4)	869 (73.6)	1181 (51.2)	0.17 (0.13–0.22, *p* < 0.001)	0.21 (0.11–0.41, *p* < 0.001)

^a^ All variables found significant in the unadjusted analysis were included in the adjusted model to identify potential predictors of intent to receive a COVID-19 vaccine.

## Data Availability

Data used in this study are available from the corresponding author on reasonable request.
